# A new process to measure postural sway using a Kinect depth camera during a Sensory Organisation Test

**DOI:** 10.1371/journal.pone.0227485

**Published:** 2020-02-05

**Authors:** Sean Maudsley-Barton, Moi Hoon Yap, Anthony Bukowski, Richard Mills, Jamie McPhee

**Affiliations:** 1 Department of Computing and Mathematics, Manchester Metropolitan University, Manchester, United Kingdom; 2 Department of Sport and Exercise Sciences, Manchester Metropolitan University, Manchester, United Kingdom; Universitat Konstanz, GERMANY

## Abstract

Posturography provides quantitative, objective measurements of human balance and postural control for research and clinical use. However, it usually requires access to specialist equipment to measure ground reaction forces, which are not widely available in practice, due to their size or cost. In this study, we propose an alternative approach to posturography. It uses the skeletal output of an inexpensive Kinect depth camera to localise the Centre of Mass (CoM) of an upright individual. We demonstrate a pipeline which is able to measure postural sway directly from CoM trajectories, obtained from tracking the relative position of three key joints. In addition, we present the results of a pilot study that compares this method of measuring postural sway to the output of a NeuroCom SMART Balance Master. 15 healthy individuals (age: 42.3 ± 20.4 yrs, height: 172 ± 11 cm, weight: 75.1 ± 14.2 kg, male = 11), completed 25 Sensory Organisation Test (SOT) on a NeuroCom SMART Balance Master. Simultaneously, the sessions were recorded using custom software developed for this study (CoM path recorder). Postural sway was calculated from the output of both methods and the level of agreement determined, using Bland-Altman plots. Good agreement was found for eyes open tasks with a firm support, the agreement decreased as the SOT tasks became more challenging. The reasons for this discrepancy may lie in the different approaches that each method takes to calculate CoM. This discrepancy warrants further study with a larger cohort, including fall-prone individuals, cross-referenced with a marker-based system. However, this pilot study lays the foundation for the development of a portable device, which could be used to assess postural control, more cost-effectively than existing equipment.

## Introduction

Postural control is key to maintaining balance during everyday activities. A decline of postural control with advancing age can cause difficulties when completing physical functional tasks and increases the risk of falls [1]. By the age of 75 years, the ability to stand on one leg with eyes closed is reduced to less than 20% of the performance of young adults [2] and the amount of postural sway during two legged quiet standing can increase by as much as 30% (eyes closed) [3]. By the time the average person reaches the age of 80 years, they are likely to fall at least once a year [4]. Falling is a cause of distress, pain, injury, loss of independence and even mortality. Although the reasons for falls in old age are varied [5], poor postural control is a key factor, which can be addressed once those at risk are identified [2]. There is a need for an inexpensive device to objectively screen for poor postural control and falls risk. In this study, we propose an analysis pipeline that could easily be incorporated into such a device.

Many existing screening tools use subjective measures, such as asking individuals or their relatives to recall their falls history through interview or questionnaire. The answers rely on the subject’s accurate recollection of past events and cannot easily identify sensory or other physiological deficits that increase falls risk [6]. An alternative to questionnaires is for a clinician to observe a set of prescribed movements [7], but this is often subjective and prone to differences between assessors in application and interpretation.

Conversely, posturography provides an objective measurement of a person’s postural sway by making postural control and balance assessments [8] based on the movement of the body’s Centre of Mass (CoM), usually calculated from quantitative ground reaction forces as measured using a force plate. The work of Nashner et al. [9], Hasselkus and Shambes [8] lead to the development of the sensory organisation tests (SOT), which is implemented in the SMART Balance Master (BM). SOTs involves static and reactive balance assessments and conditions that place emphasis on visual (eyes open/closed), vestibular, or proprioceptive afferents that govern postural control.

The Balance Master’s SOT was selected because it is regarded as a valid tool to investigate different aspects of balance [10–12] including falls risk amongst older adults [13–15], proprioceptive decline [16], the effects of age and gender on postural control [17], and the effectiveness of balance-based exergaming [18]. However, high costs and low availability of the specialist equipment needed for posturography and SOT means they are not practical for wide-scale screening [2].

The Kinect depth camera offers the potential to accurately and reliably assess many aspects of human movement. However, their use as a posturographic device is underexplored. They are affordable, portable and can potentially be used in a wide range of home or clinical settings. Previous studies [19–23] have considered the use of Kinect as a way to replace marker-based systems (e.g. Vicon, Qualisys). The near universal conclusion of these studies is that Kinect can be considered equivalent to the marker-based systems. However, the use of depth cameras to assess postural sway as an indicator of postural control is rarely considered. Our proposed approach addresses this gap. It seeks to measure postural control by tracking CoM in an equivalent fashion to the way it is measured by the most widely used means of assessing standing postural control, i.e. force plates. Force plates are used in the assessment and diagnosis of many conditions, including falls risk [24, 25] and it is with force plates that the Balance Master measures sway.

Yeung et al. [26] did consider Kinect’s use in posturography. The authors outline an approach that uses Kinect to calculate the Total Body Centre of Mass (TBCM) by segmenting the body, as described by Dempster [27]. The CoM is calculated for each segment and TBCM is then calculated as a weighted average of the CoM of all segments, using Eq 1.

$$\begin{array}{c} \begin{array}{c} {}^{x}TBCM=\frac{\sum m_{i}x_{i}}{M}\\ {}^{y}TBCM=\frac{\sum m_{i}y_{i}}{M} \end{array} \end{array}$$ (1)

where *^x^TBCM*,^*y*^*TBCM* are coordinates of *TBCM*; *x*_*i*_, *y*_*i*_ are coordinates of the i-th segment; *m*_*i*_ is mass of the i-th segment and *M* is the total body mass of the segment body model. The authors concluded that Kinect is an excellent tool for measuring TBCM.

In the current study, we demonstrate a much simpler method of calculating CoM, first used by Leightley et al. [28], is able to achieve similar results. Leightley’s method takes the euclidean mean of 3, well-tracked joints (hip left, hip right, spine mid) to be a good estimate of the CoM position. Previous studies [26, 29] have demonstrated that the accuracy of Kinect’s joint tracking is related to the angle between the Kinect and the joint. This means that ankle and foot joints are tracked very poorly. Joints which have a less steep angle to the Kinect (e.g. the hip joints) are tracked with high accuracy. Poor tracking of joints can cause issues when estimating the TBCM, an issue which Leightley’s method avoids. The human skeleton can be considered as a chain of connected joints meaning the positions of knee, ankle, and foot joints affect the CoM position without the need to consider them directly. Thus for an upright stance, the lengthy calculation of TBCM is not required for our application.

The aims of this study are: (1) develop a pipeline to track CoM and calculate postural sway from the output of a Kinect camera and (2) compare the output of the pipeline to the SMART Balance Master.

## Materials and methods

### Participants

This study was approved by the Manchester Metropolitan University Research Ethics Committee. All participants provided written informed consent. Fifteen injury-free individuals (mean ± SD age: 42.3 ± 20.4 yrs; height: 172 ± 11cm; weight: 75.1 ± 14.2kg; BMI: 25.3 ± 3.3 kg/m^2^; male = 11) took part in 346 trials during completion of the six components of the SOT used by the SMART Balance Master (NeuroCom International, USA), to assess postural sway during static and dynamic challenges. We chose a wide age range to ensure a wide range of postural sway was recorded. Postural sway is known to increase with age, as part of the normal ageing process. [3]. The age profile, of the participants, was 6 young (20-30), 5 middle age (31-59) and 4 older (>60).

**Fig 1:**
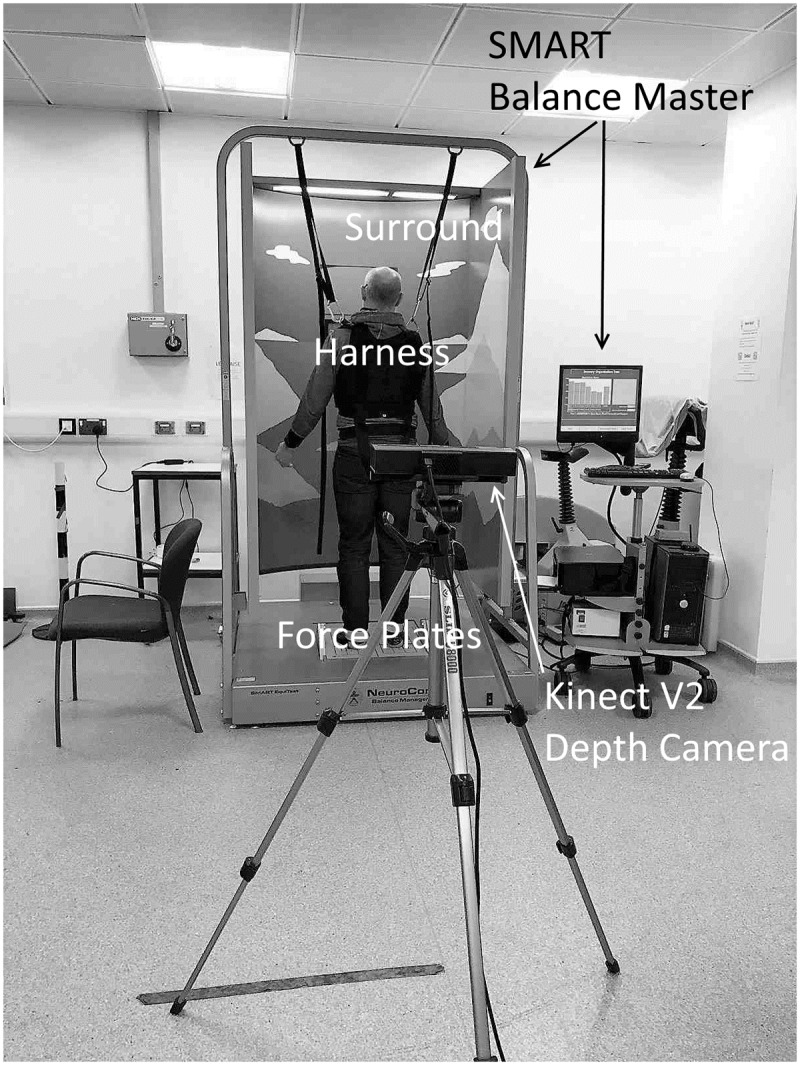
Setup of Balance Master and Kinect V2, depth camera.

The individual, pictured in Fig 1 has given informed consent for the use of their image, as outlined in PLOS consent form.

For this pilot study, no individuals with a history of falls were included. Also, several participants took part in more than one set of trials. This is a valid choice, as this is a study of agreement between two methods, not an investigation to identify those with balance impairment.

### Procedure

The participants were simultaneously recorded using the EquiTest software that comes bundled with the SMART Balance Master and the CoM path recorder. CoM path recorder is custom software, detailed in section Recording of CoM path, using CoM path recorder. It processes the output of the Kinect depth camera into a 2D CoM path. Participants performed the six components of the SOT while standing, on the force plates incorporated into the Balance Master. The Balance Master was controlled and data recorded using the EquiTest software. The Kinect was controlled using the CoM path recorder. Participants wore a safety harness throughout all assessments to prevent falls. All six components of the SOT (outlined below) were carried out in accordance with the Balance Master operator instructions. The instructions require participants to stand on two legs approximately shoulder-width apart with heels aligned to markers on the force plates [30].

The six components of the SOT are as follows: (a) eyes open, platform fixed; (b) eyes closed to remove visual input; (c) eyes open with moving surround, to create sensory conflict between visual input (simulating a moving room) and vestibular inputs (a stable room); (d) eyes open and the platform support rotating freely to disrupt somatosensory and proprioceptive feedback from the feet and ankles; (e) eyes closed and the platform support rotating freely; and (f) eyes open with moving surround and the platform support rotating freely.

Two consistent trials, for each condition, were included in this study. Inconsistent trials and fails were excluded from further analysis. All assessments were conducted in the sequence of (a) to (f), as recommended by the operator instructions, this increases difficulty progressively. Each trial (an instance of an individual, carrying out one aspect of the SOT), was repeated twice, except if the second trial was inconsistent with the first, or was marked as a fail, in which case the participant was allowed a third attempt. A trial was marked as a fail if a participant touched the upright supports on the Balance Master frame or relied on the safety harness to maintain an upright posture for any reason.

### Experimental setup

Participants stood upright on the force plates of the Balance Master, facing towards the large surround approximately 1m away. The surround is used to create visual-vestibular conflict, but obscured the front view of the participant (Fig 1). Therefore, the Kinect was positioned to capture the rear-view of the participant 2.5 m from the participant at a height of 1.2 m from the floor. The distance was selected after pilot trials to confirm that people of all heights could be captured equally well while their feet were placed correctly, along the foot markers on the force plates (Fig 1).

### Recording of CoM path

#### Recording of CoM, using SMART Balance Master

**Fig 2:**
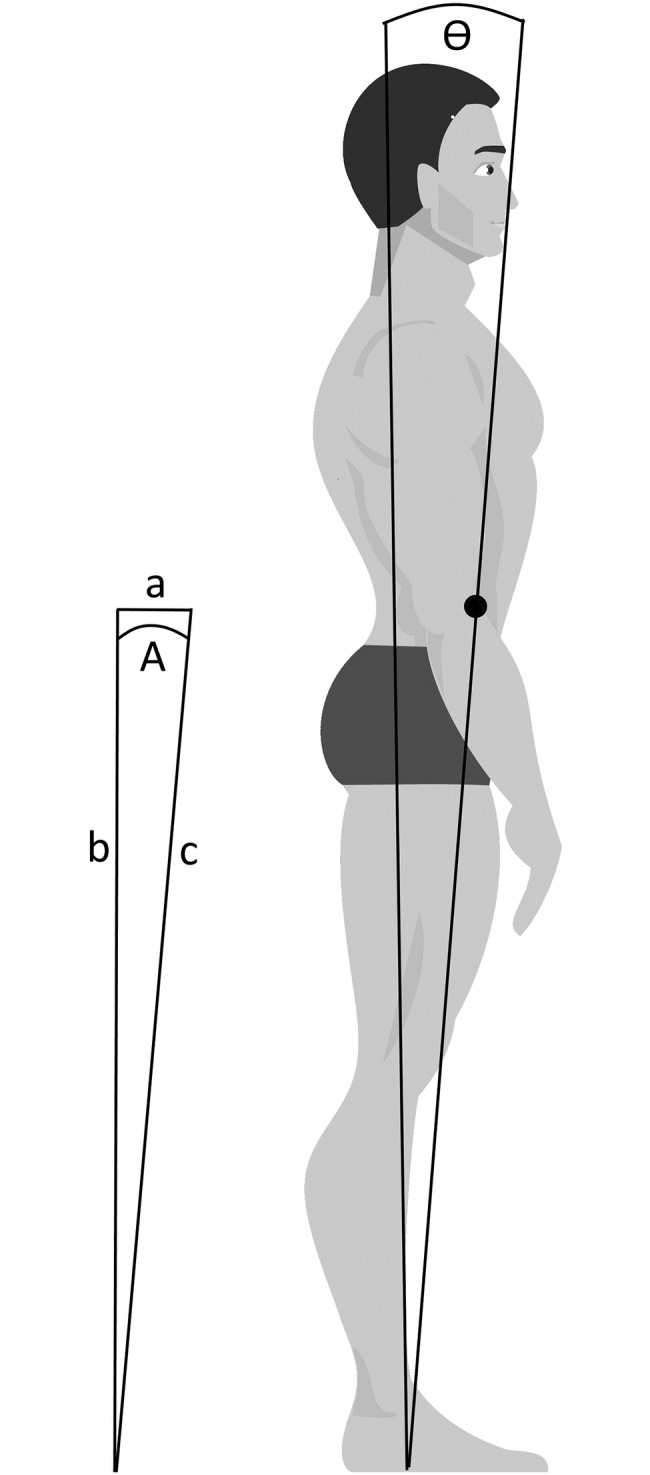
Diagram of sway angle calculation used by the Balance Master [30].

The Balance Master [30] estimates a vertical projection of the Centre of Mass (CoM) from Centre of Force (CoF) data using the method described by Morasso et al. [31]. This method assumes that the body is rigid and the CoF is mid-way between the two feet with a single pivot at the ankle (Fig 2). The vertical projection of the CoM is estimated to be 0.5527 of the person’s height (represented by length **c** in Fig 2). The value for **a** is obtained by taking the CoF value from the force plates and inclining it by -2.3°, estimated to be the average anterior lean when standing. The force plates have a sampling rate of 100 Hz.

The CoM path was recorded using the EquiTest software, bundled with the SMART Balance Master. The CoM path is plotted in two dimensions, mediolateral and anterior-posterior.

#### Recording of CoM path, using CoM path recorder

Kinect measures the distance from the participant to the camera in three dimensions, using the time-of-flight of an infrared beam, at a rate of 30 Hz. From this information, Kinect fits a human skeleton to a 25-joint model [32], which has very high agreement with skeletons generated from marker-based systems [26].

**Fig 3:**
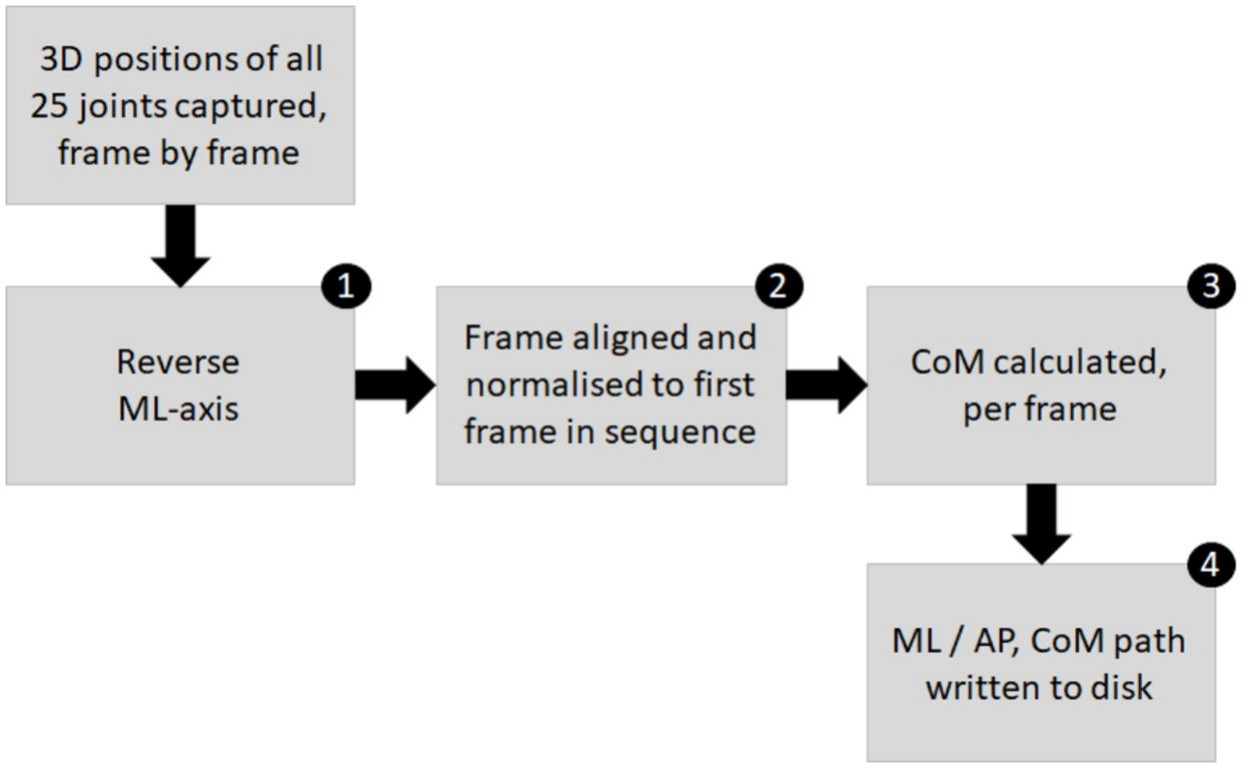
The pipeline of the CoM path recorder.

The CoM path recorder is custom software, written in C# using Visual Studio and the Kinect SDK 2.0. It takes a series of skeleton frames and derives a CoM path. The pipeline of the CoM path recorder is shown in Fig 3. The steps of the pipeline are as follows: 1) The ML-axis of the skeletons are reversed, to take into account the rear position of the Kinect camera; 2) Each skeleton frame, is aligned to the first frame of the recording, making all subsequent movements relative to this initial position [33]; 3) The position of CoM is estimated, as described, in the section Frame-wise calculation of CoM; and 4) The ML and AP elements of the CoM path to disk.

**Fig 4:**
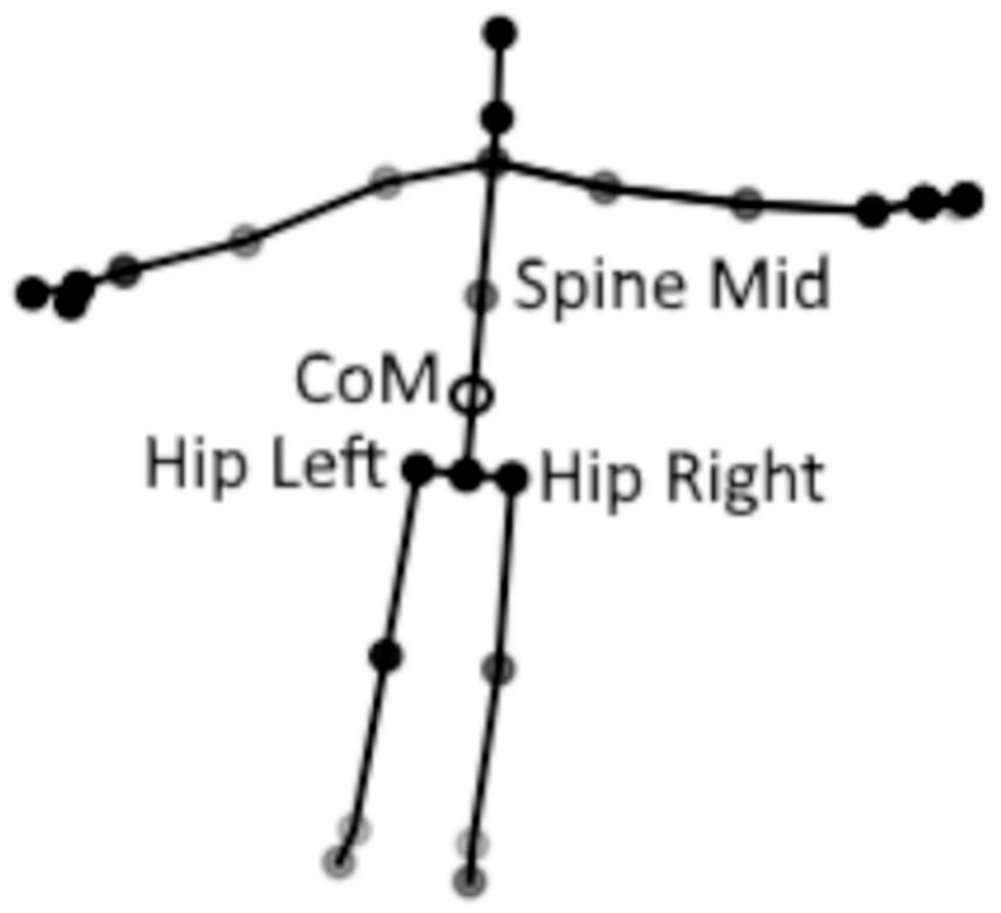
Kinect V2 skeleton, the joints used to estimate CoM and the CoM position, are labelled.

**Frame-wise calculation of CoM** The position of CoM was calculated, frame-by-frame by taking the euclidean average of the left-hip, right-hip and mid-spine joints, as defined by Eq 2, first used by Leightley et al. [28]. This method estimates the position of CoM in three dimensions without needing to rely on the assumptions made by the Balance Master.

$$\begin{array}{c} \begin{array}{c} CoM_{ML}=\frac{J1_{ML}+J2_{ML}+J3_{ML}}{3}\\ CoM_{AP}=\frac{J1_{AP}+J2_{AP}+J3_{AP}}{3}\\ CoM_{SI}=\frac{J1_{SI}+J2_{SI}+J3_{SI}}{3} \end{array} \end{array}$$ (2)

where J1 = Hip left, J2 = Hip right, J3 = Spine mid (see Fig 4 for details of joint position).

### Creation of CoM time series

As noted by Prieto et al. [34], when calculating sway, precise foot placement is difficult. This makes meaningful comparison between individuals difficult, the same can be said for the comparison of methods. A more robust approach is to calculate a time series that places the mean position of the overall movement at the origin. This is achieved by subtracting the mean position in the ML and AP direction from each step in the time series (Eq 3). The resultant times series (RD) was used for the comparison of the two methods.

$$\begin{array}{c} RD=\sqrt{\left[{\left(ML_{i}-\bar{ML}\right)}^{2}+{\left(AP_{i}-\bar{AP}\right)}^{2}\right]} \end{array}$$ (3)

### Calculation of sway

The resultant time series were used to calculate RMS of sway measured by each method, using Eq 4, where *RD* is the time series calculated in Eq 3 and N is the number of time points in the time series.

This measure of postural sway calculates the average deviation from the mean position, assuming the participant is standing upright [34, 35]. MATLAB 2019a was used to implement Eqs 3 and 4

$$\begin{array}{c} swayRMS=\sqrt{\frac{\sum_{i=1}^{N}RD^{2}}{N}} \end{array}$$ (4)

### Data exclusions

A total of 56 recordings were removed for various reasons, as detailed in Table 1. The remaining 288 records were used in the analysis.

**Table 1:** Table of exclusions.

Reason for exclusion	Description	#
Extra recordings	For each trial, if a participant did not complete two consistent trials, they were offered a third trial. Only the two most representative trials were used.	29
The participant fell	The participant fell while attempting a trial.	3
Recordings out-of-sync	The start of the Kinect recording was not coincident with the start of the balance master recordings.	14
Over-recording of a previous trial	One trial was recorded over another trial.	6
Malformed skeletons	Kinect was not able to track all the joints consistently during the recording.	5
The harness caused confusion	Kinect mistook the harness for a limb.	1
	Total	58

### A priori sample size calculation

A priori sample size estimation was carried out to ensure there was enough power to detect differences between the two methods. We utilised the recordings we made while experimenting with the best position for the Kinect camera. Using the mean and standard deviation of this data, we calculated the sample size required for each trial, using G*Power. The results are shown in Table 2, along with the actual sample size used for analysis.

**Table 2:** A priori power calculations G*Power was used to calculate the sample size required for 95% power. The data came from an initial study, used to ensure the placement of the Kinect camera was correct.

	Sample size @0.95 power	Actual Sample Size
**a) Quiet standing eyes open**	12	44
**b) Quiet standing eyes closed**	11	48
**c) Surround moving eyes open**	29	50
**d) Support moving eyes open**	45	50
**e) Support moving eyes closed**	5	48
**f) Support & surround moving eyes open**	11	48

### Data analysis

The main analysis used in this study was the Bland-Altman test for agreement between methods [36]. In addition, several supporting analysis were carried out.

The results obtained from each method were assessed for normality using D’Agostino-Pearson and Shapiro–Wilk methods. The difference between each method was normally distributed (Table 3). However, the range of values produced by each method was found to be non-normal. Bland and Altman noted that this is often the case [36]. Normality was calculated using the scipy python library.

**Table 3:** The normality of the difference between the two methods given by D’Agostio-Pearson and Shapiro-Wilk tests for normality.

	D’Agostino-Pearson *α* = 0.05	p	Shapiro–Wilk *α* = 0.05	p
**a) Quiet standing eyes open**	Yes	0.826	Yes	0.689
**b) Quiet standing eyes closed**	Yes	0.157	Yes	0.256
**c) Surround moving eyes open**	Yes	0.275	Yes	0.229
**d) Support moving eyes open**	Yes	0.100	Yes	0.154
**e) Support moving eyes closed**	Yes	0.171	Yes	0.204
**f) Support & surround moving eyes open**	Yes	0.406	Yes	0.297

One-sample t-tests were used to provide a significance value for the absolute agreement between methods, i.e. an hypothesised difference of zero. The t-tests were carried out using SPSS (v. 21. IBM, US). Significance was accepted at p<0.05.

The repeatability of each method was assessed by comparing the repeated measures. Standard deviation (SD) and coefficient of repeatability (CR) were calculated for each method (Table 4).

**Table 4:** Repeatability of each method, Balance Master (BM) and the Proposed Pipeline (PP), has measured by the Standard Deviation (SD) and Repeatability Coefficient (CR).

	Method	SD (mm)	95% CR
**a) Quiet standing eyes open**	PP	**0.85**	**2.35**
	BM	**0.88**	**2.45**
**b) Quiet standing eyes closed**	PP	0.84	2.33
	BM	0.72	2.00
**c) Surround moving eyes open**	PP	**1.19**	**3.31**
	BM	**1.14**	**3.16**
**d) Support moving eyes open**	PP	**1.50**	**4.17**
	BM	**1.49**	**4.15**
**e) Support moving eyes closed**	PP	3.40	9.41
	BM	2.86	7.93
**f) Support & surround moving eyes open**	PP	7.37	20.43
	BM	7.94	22.00

**Fig 5:**
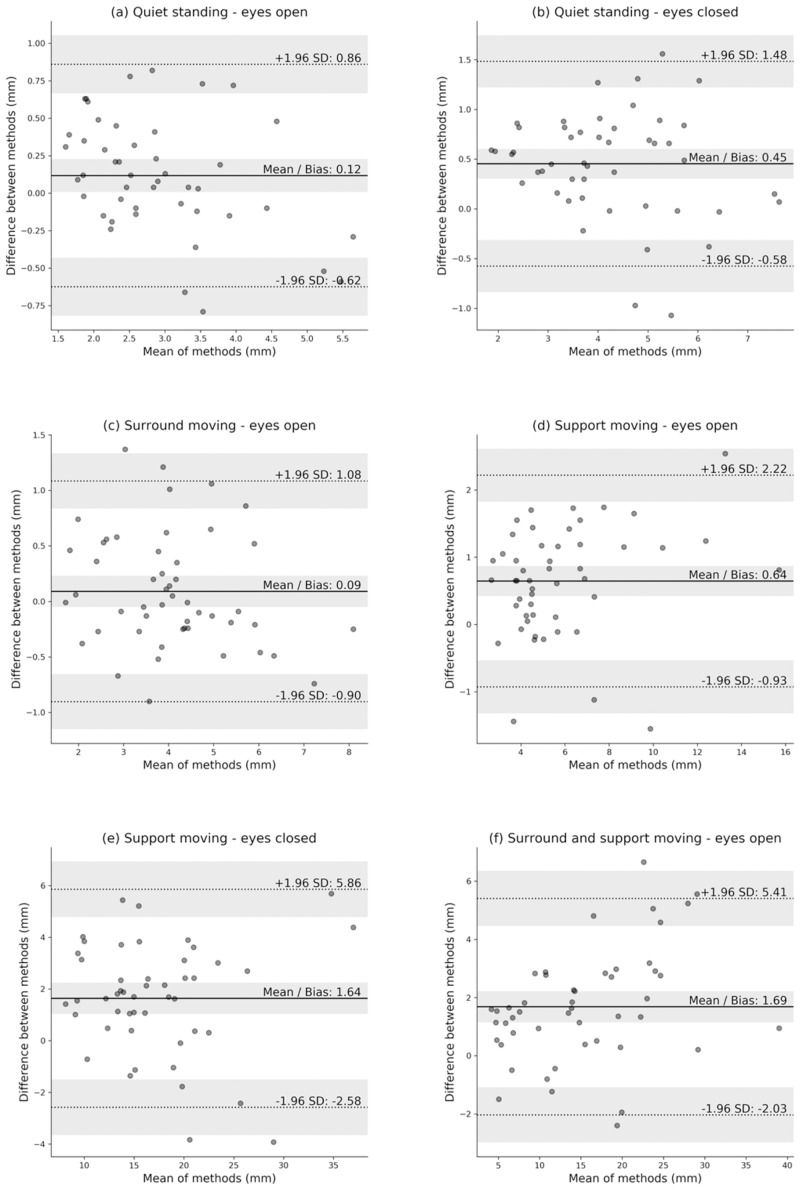
Bland-Altman plot of Balance Master vs the Proposed Pipeline’s estimates of postural sway. Bold horizontal line indicates the mean, dashed horizontal lines indicate two standard deviations from the mean. The shaded areas represent associated confidence intervals. The figures marked a-f represent the six conditions of the SOT test.

Bland-Altman plots were created (Fig 5), using all available data for each method, without averaging over repeated measures. Repeatability and Bland-Altman tests were carried out using the Analyse-it plugin for excel (v. 5.40.2).

Additionally, descriptive statistics were calculated using SPSS (v. 21. IBM, US).

## Results

Postural sway measured by the proposed pipeline did not differ significantly from that measured by the Balance Master, for eyes open conditions with a firm support. All other conditions showed significant disagreement. The disagreement, expressed as bias, increased with increasing challenge.

### Repeatability

Repeatability, also known as Precision, was calculated for each method. Both the proposed pipeline and the Balance Master, show increasing variability with increasing balance challenge. With eyes open conditions showing the best agreement (Table 4).

### Agreement of postural sway measurement

One-sample t-tests were carried out on the differences between sway calculated by each method. Conditions (a) quiet standing, eyes open and (c) surround moving, eyes open showed no significant difference between methods. For all other conditions, a significant difference was found. Significance was accepted with an *α* of 0.05 (Table 5). NB As an alternative approach, we used the non-parametric, Wilcoxon Signed Rank test (designed for non-normal data). We used matched pairs of results from the two methods (which had been shown to be non-normal). Wilcoxon Signed Rank test produced the same result as the one-sample t-test.

**Table 5:** A summary of the agreement of postural sway derived from the two methods: Balance Master (BM) and the Proposed Pipeline (PP). The mean—within each method, mean—between the methods (bias), the 95% Confidence Interval (CI) and Limits of Agreement (LOA) and the significance of the t-test are shown.

	BM mean sway (mm)	PP mean sway (mm)	Mean difference BM-PP (bias) (mm)	95% CI for Bias	LOA	t-test (p)
**a) Quiet standing eyes open**	2.96 ±0.94	2.84 ±1.10	**0.12** ±**0.38**	0.004 to 0.232	-0.62 to 0.86	**0.161**
**b) Quiet standing eyes closed**	4.45 ±1.36	3.99 ±1.45	0.45 ±0.53	0.300 to 0.609	-0.58 to 1.48	3.39E-07
**c) Surround moving eyes open**	4.10 ±1.35	3.99 ±1.46	**0.09** ±**0.50**	-0.056 to 0.236	-0.90 to 1.08	**0.211**
**d) Support moving eyes open**	6.12 ±2.84	5.53 ±2.68	0.64 ±0.82	0.414 to 0.876	-0.93 to 2.22	9.10E-07
**e) Support moving eyes closed**	17.93 ±6.29	16.29 ±6.37	1.64 ±2.18	1.007to 2.270	-2.58 to 5.85	3.99E-06
**f) Support & surround moving eyes open**	16.12 ±8.51	14.43 ±7.79	1.69 ±1.63	1.130 to 2.244	-2.03 to 5.41	1.93E-07

Bland-Altman plots (Fig 5) were used to assess the agreement between the two methods. The agreement results are summarised in Table 5. A small disagreement (bias), around 0.1mm, was seen for the conditions that showed no significant difference in calculated sway (a and c). However, as the balance challenge increases, the disagreement between the two methods increases, the largest bias being 1.69mm.

### Implications of the increased disagreement

The eyes open condition show the most similarity in repeatability. Looking at the bias between the two samples, conditions where the participant is standing on a firm surface, with eyes open agree the best. However, as the balance challenged increases, either by removing vision or by perturbing balance by standing on a pivoting platform, the two results, increasingly disagree.

The differences, seen in these results, may be explained by the fundamentally different approaches each method takes to estimate the CoM position, are discussed in the following section.

## Discussion

In this study, we propose a pipeline that is able to assess upright human postural sway. It makes use of an inexpensive and portable depth camera (Kinect V2), in combination with custom software that calculates CoM directly from skeleton joints. We also carried out a pilot-study that compares the postural sway calculated from the proposed pipeline and a Balance Master, obtained during a Sensory Organisation Test (SOT).

We examined the repeatability of each method (Table 4), i.e. the agreement between repeated measures. The comparison was based on the (SD) and reliability coefficients (CR), for each method. Both methods show an increase in variability with task difficulty. The SOT test uses this variability to identify balance defects. In the SOT, the ratio of sway measured in quiet standing eyes closed (b) vs quiet standing eyes open (a) is used as a measure of the reliance on the somatosensory system to balance. This is also known as the Romberg Ratio. The reliance on the visual system is given by the ratio of support moving, eyes open (d) vs quiet standing eyes open (a) (the measures with the greatest similarity in the repeatability test) and the reliance on the vestibular system is given by support moving eyes closed (e) vs quiet standing eyes open (a). In all these assessments, quiet standing eyes open (a) is used as a baseline measure [30]. This matches the intuition that in a given population, the ability to balance with eyes open is essential and so well-practised. However, the ability to balance well, when challenged in unfamiliar ways produces a wider range of scores, seen as increasing variance.

We further examined the agreement between the two methods using Bland-Altman plots (Fig 5), and one-sample t-tests, with an hypothesised, mean difference of zero. The plots show the mean difference between measures (bias) is smallest for the most every-day tasks (eyes open with the least challenge), but bias increases with increasing task difficulty. The t-test suggests that the two methods only agree well for eyes open conditions with a firm surface. To understand how these disagreements may occur, it is worthwhile considering two elements. 1) the way the human body reacts to quiet standing vs its reaction to perturbation. Winter in his review on human balance [37] noted that the human body pivots about the ankle (the ankle strategy) in quiet stance and about both hip and ankle in reaction to a perturbation (the hip strategy), such as standing on a pivoting platform. The Balance Master uses a pivoting platform to induce perturbation in the tests which generated the biggest disagreement between methods (d to f). The induced perturbation causing an increase in postural sway amplitude. Black et al. [38] noted that quite standing with eyes closed also increases postural sway amplitude, and so a switch to a hip strategy, for some people. In condition (b) quiet standing with eyes closed, we see an increased bias between, compared to condition (a), although the increase is less than for conditions d-f, where the pivoting platform induces a greater postural sway. These observations lead to the second point. 2) The way the two methods estimate CoM is quite different. The Balance Master uses the most common method of estimating CoM, when using force plates, the inverted pendulum model, which ignores the hip and knee joints. In order to estimate the position of the CoM, using this method, an average value for the static incline of the body and an average offset from the position of the CoF, proportional to a person’s height, is used to relate the CoF to the CoM [30]. The proposed pipeline calculates CoM from the Kinect data, as described in Eq 2; its estimate of CoM relates directly to the skeletal structure. Although it uses the values of only 3 joints (left hip, right hip and spine mid), these joints do not exist in isolation. Their movements are influenced directly by the movements of other anatomical structures such as the ankle, knee and hip joints, as well as the spine, arms and head. Previous reports questioned the assumptions used routinely to estimate CoM from CoF data. For example, Cretual et al. [39] suggested the single pendulum model should be used with caution to estimate CoM during more challenging conditions. Lafond et al. [40] also found error in this method of calculating CoM for more difficult poses, and Yeung et al. [26] demonstrated that Kinect performed better when recording more challenging balance tasks compared with force plates. Benda et al. [41] demonstrated that the accuracy of CoM estimated from CoF reduces with increased dynamics. Although the literature may go some way to explain the disagreement between the two methods, future work is warranted to empirically, demonstrate the reasons for the differences. This future work should provide a three-way cross-validation between CoM, measured using the proposed pipeline, a high quality marker-based system and a high quality force plate. Separately, future work should examine the potential of the proposed pipeline in the identification of individuals with balance impairments.

For now, we can say that the proposed pipeline shows no significant difference to the Balance master when measuring sway for quiet standing, eyes open and quiet standing with a moving surround, eyes open. Quiet standing with a moving surround, eyes open is designed to assess individuals with a vestibular defect. Their over reliance on the visual system, inducing a substantial increase in postural sway. Since all our participants were healthy, an ankle strategy is sufficient to maintain balance, for both these conditions.

This study was designed as a proof of concept and shows that assessment of postural control by depth camera is worth pursuing. Especially for applications where devices, such as the Balance Master, are too expensive or too cumbersome to be practical.

### Limitations, considerations and future work

(1) Our assessments were completed in laboratory conditions. In more informal settings, there is the potential for Kinect to confuse non-human elements, such as table and chair legs for human limbs. (2) The current study only includes healthy individuals. Future work should extend these initial findings, to a larger group, including individuals who suffer from recurrent falls. (3) In this study, we used the Balance Master to automate the SOT. The Balance Master uses pivoting force plates and a pivoting surround to produce challenging balance conditions. In order to further the cause of machine-based balance assessments in informal settings, future work will need to utilise more portable means of challenging balance. These include compliant foam pads and visual conflict domes. For instance, the Clinical Test of Sensory Integration and Balance (CTSIB) [42] uses these items to replicate the SOT test, without the need for costly equipment. (4) Balance Master’s force plates are not as accurate as more modern designs. Future work should incorporate the newer plates, ideally as part of a three-way validation with a marker-based system.

## Conclusion

In this study, we propose a novel pipeline to assess upright postural sway. We carried out a pilot study to compare the results of the proposed pipeline to results from a Balance Master, obtained from simultaneously testing 15, healthy individuals (age: 42.3 ± 20.4 yrs, height: 172 ± 11 cm, weight: 75.1 ± 14.2 kg, male = 11). Our initial findings suggest that the methods agree well for static assessments of balance, with eyes open, but the agreement reduces under more challenging conditions. That said, the new method warrants further investigation, with a wider variety of devices and a larger cohort, including people for who falling is an ongoing issue.
